# Future Aspects of CDK5 in Prostate Cancer: From Pathogenesis to Therapeutic Implications

**DOI:** 10.3390/ijms20163881

**Published:** 2019-08-09

**Authors:** Muhammet Oner, Eugene Lin, Mei-Chih Chen, Fu-Ning Hsu, G M Shazzad Hossain Prince, Kun-Yuan Chiu, Chieh-Lin Jerry Teng, Tsung-Ying Yang, Hsin-Yi Wang, Chia-Herng Yue, Ching-Han Yu, Chih-Ho Lai, Jer-Tsong Hsieh, Ho Lin

**Affiliations:** 1 Department of Life Sciences, National Chung Hsing University, Taichung 40227, Taiwan; 2 Department of Urology, Chang Bing Show Chwan Memorial Hospital, Changhua 505, Taiwan; 3 Translational Cell Therapy Center, Department of Medical Research, China Medical University Hospital, Taichung 40447, Taiwan; 4 Division of Urology, Department of Surgery, Taichung Veterans General Hospital, Taichung 40705, Taiwan; 5 Division of Hematology/Medical Oncology, Department of Internal, Medicine, Taichung Veterans General Hospital, Taichung 40705, Taiwan; 6 Division of Chest Medicine, Department of Internal Medicine, Taichung Veterans General Hospital, Taichung 40705, Taiwan; 7 Department of Nuclear Medicine, Taichung Veterans General Hospital, Taichung 40705, Taiwan; 8 Department of Surgery, Tung’s Taichung Metro Harbor Hospital, Taichung 435, Taiwan; 9 Department of Physiology, School of Medicine, Chung Shan Medical University, Taichung 40201, Taiwan; 10 Department of Microbiology and Immunology, Chang Gung Medical University, Taoyuan 33302, Taiwan; 11 Department of Urology, University of Texas Southwestern Medical Center, Dallas, TX 75390, USA; 12 Program in Translational Medicine and Rong Hsing Research Center for Translational Medicine, National Chung Hsing University, Taichung 40227, Taiwan

**Keywords:** cyclin-dependent kinase 5 (CDK5), p35, androgen receptor (AR), prostate cancer

## Abstract

Cyclin-dependent kinase 5 (CDK5) is a unique member of the cyclin-dependent kinase family. CDK5 is activated by binding with its regulatory proteins, mainly p35, and its activation is essential in the development of the central nervous system (CNS) and neurodegeneration. Recently, it has been reported that CDK5 plays important roles in regulating various biological and pathological processes, including cancer progression. Concerning prostate cancer, the androgen receptor (AR) is majorly involved in tumorigenesis, while CDK5 can phosphorylate AR and promotes the proliferation of prostate cancer cells. Clinical evidence has also shown that the level of CDK5 is associated with the progression of prostate cancer. Interestingly, inhibition of CDK5 prevents prostate cancer cell growth, while drug-triggered CDK5 hyperactivation leads to apoptosis. The blocking of CDK5 activity by its small interfering RNAs (siRNA) or Roscovitine, a pan-CDK inhibitor, reduces the cellular AR protein level and triggers the death of prostate cancer cells. Thus, CDK5 plays a crucial role in the growth of prostate cancer cells, and AR regulation is one of the important pathways. In this review paper, we summarize the significant studies on CDK5-mediated regulation of prostate cancer cells. We propose that the CDK5–p35 complex might be an outstanding candidate as a diagnostic marker and potential target for prostate cancer treatment in the near future.

## 1. CDK5 and the Nervous System

Cyclin-dependent kinase 5 (CDK5) is an essential member of the proline-directed serine/threonine kinase family [1]. CDK5 does not directly participate in the cell cycle process itself; instead, CDK5 regulates the cell-cycle-related proteins [2]. CDK5 is activated by its regulatory subunits—including p35, p25, and p39—which are mainly expressed in postmitotic neurons according to previous findings [3,4,5,6]. The CDK5–p35 complex has essential roles in the regulation of central nervous system (CNS) development [2,7] and neurodegenerative diseases [8,9]. Our previous study demonstrated that the CDK5–p35 complex could be regulated by nerve growth factor signaling in neuronal differentiation, and the cAMP-related pathway plays an alternative role in this regulation [10]. Physiologically, CDK5 has been found to regulate the neuronal cytoskeleton [11], neuronal migration [12], synaptic function [13], neuronal differentiation, neurite length and complex cognitive functions [2,14] in the developing brain [15]. CDK5 is also important for the regulation of dendrite development in the primary culture of rat neurons [16]. Additionally, CDK5 contributes to neurodegenerative diseases such as Alzheimer’s disease (AD). CDK5 hyperactivation leads to neuronal death under the accumulation of amyloid β peptides [17] and is widely believed to act as molecular pathogenesis of neurodegenerative diseases. Our previous study also demonstrated the essential role of CDK5 under Abl regulation using a *Drosophila* neurodegenerative model [18]. Inhibition of overactivated CDK5 showed a neuroprotective effect against neurodegenerative diseases in a zebrafish model [11]. Besides, the blocking of CDK5–p25 interaction decreased CDK5 activation, and notably, reduced tau protein phosphorylation and accumulation, which is an important factor in the neuropathology of AD [19].

## 2. CDK5 and Androgen Production

Increasing lines of evidence suggest that CDK5 has various extra-neuronal roles [20]. CDK5 is essential for the regulation of insulin secretion in pancreatic β cells [21]. Moreover, CDK5 has been associated with obesity-linked insulin resistance and regulates diabetes-responsible gene expression in adipose tissues [22]. In addition to insulin secretion and metabolic issues, recent studies have demonstrated that CDK5 is significantly associated with androgen production. CDK5 and p35 expression have been identified in the male reproductive system [23,24]. To clarify the regulatory role of CDK5 and p35 in male reproduction and understand the relationship between CDK5 and prostate cancer, we demonstrated that human chorionic gonadotrophin (hCG), which is involved in major reproductive processes, regulates CDK5–p35 activity in rodent Leydig cells [25]. Leydig cells are responsible for androgen production in the male reproductive system. Blocking of CDK5 activity resulted in decreased production of testosterone in rodent Leydig cells. Moreover, CDK5 activity plays an essential role in androgen steroidogenesis through regulating steroidogenic acute regulatory (StAR) protein in mitochondria of Leydig cells, which is the rate-limiting step for androgen production. StAR protein has also been shown to be involved in some pathological events, such as obesity and testicular cancer [26,27,28]. CDK5 phosphorylates StAR protein and increases its protein stability. Therefore, CDK5-dependent regulation of StAR protein is responsible for maintaining the level of StAR protein and promoting daily androgen production. Our previous study was the first to demonstrate that CDK5–p35 complex plays an essential role in regulating androgen production in rodent Leydig cells through post-translational modification of StAR protein, although the phosphorylation site is still unclear. Thus, CDK5 and p35 are essential proteins in male reproduction, and the interaction between CDK5–p35 and StAR protein might be a potential monitoring target in androgen-related diseases, which is an important issue for prostate cancer.

## 3. The Androgen Receptor and Prostate Cancer

The androgen receptor is a ligand-dependent transcriptional regulatory protein, which belongs to the category of nuclear steroid receptor, the largest eukaryotic transcription factor family [29]. In healthy prostatic epithelium tissue, AR, as a transcription factor, plays an essential role in regulating terminal differentiation, suppression of apoptosis, and hormone secretion [30]. The activation of AR by binding to androgens is associated with sexual reproduction and pubertal changes while maintaining libido and spermatogenesis levels in adult males [31,32,33,34,35,36]. AR in healthy prostate epithelium tissue regularly controls cell proliferation and programmed cell death; however, the loss of this control mechanism is observed in prostate cancer cells, and the molecular mechanisms remain unclear [31]. AR signaling plays a crucial role in cell proliferation, survival, and invasion in prostate cancer development [31]. Androgen, including testosterone and dihydrotestosterone, activates AR, and regulates its biological functions. Androgen is primarily produced by the Leydig cells in the testes, as described in the previous paragraph [37]. The classical AR transactivation process is initiated by the binding of androgen to the ligand binding domain (LBD) of AR and triggers AR dimerization, phosphorylation, as well as conformational change. Subsequently, the dimerized AR translocates into the nucleus and binds to the androgen responsive element (ARE) of target genes to promote downstream gene expression [31,38,39]. Alternatively, AR can also be regulated by different signaling pathways such as MAPK/ERK, AKT [30,31,39], PI3K/AKT/mTOR [40,41,42], and WNT signaling in the development and tumorigenesis of prostate cancer [43]. Besides, ligand-independent activation of AR could be modulated by the involvement of growth factors [31,44,45]. Regulation of AR can be controlled by post-translational modifications such as acetylation, methylation, ubiquitylation, and phosphorylation [46]. Phosphorylation of AR on serine or tyrosine residues is correlated with various biological processes such as transcriptional regulation, activation, degradation, or prostate cancer growth [47]. It has been estimated that there are 40 putative phosphorylation sites on AR [48]. Several studies have reported that the phosphorylation of AR at serine or tyrosine sites changed AR function and the activation of AR-related gene expression [40,49,50]. AR Ser-81 phosphorylation shows the highest basal level responding to androgen binding [40,51]. Protein Kinase C (PKC) has the consensus sequence of phosphorylation around AR Ser-81 and is capable of regulating AR transactivation [51]. Recent studies have demonstrated that stromal-cell-derived factors phosphorylate AR Ser-81 and regulate the proliferation of prostate cancer by activating the ERK pathway [52]. After our first demonstration of CDK5 in prostate cancer cells [53], we also showed that CDK5 phosphorylates AR at Ser-81 in prostate cancer cells and regulates the stabilization of AR proteins, therefore, the prostate tumor growth was promoted [40]. Based on the multiple roles of AR in prostate cancer, CDK5 might be one of the relevant regulators in the cancer progression; this is discussed in the following section.

## 4. CDK5 and Prostate Cancer

**Figure 1:**
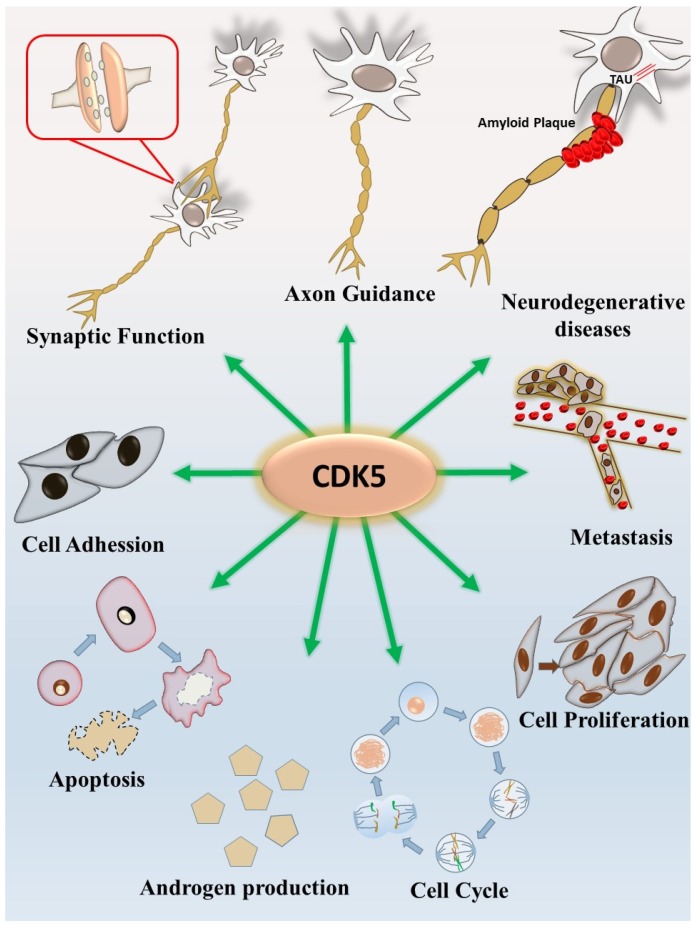
A summary of the various cyclin-dependent kinase 5 (CDK5)-mediated biological processes. CDK5 plays important roles not only in the central nervous system but also in different biological processes. Functions in the central nervous system include synaptic function, axon guidance, cell adhesion, and neurodegenerative diseases. Functions outside of the central nervous system include androgen production, cell cycle, cancer cell proliferation/apoptosis, and tumor metastasis.

It is believed that CDK5 promotes self-renewal of malignant brain tumor stem cells such as glioma stem cells [54]. Bioinformatic analyses have demonstrated that increased CDK5 expression importantly supports the migration, proliferation, and metastasis of human lung cancer [55]. One study demonstrated that CDK5 promotes DNA replication due to stress checkpoints, and therefore contributes to the metastasis of breast cancer [41]. Overexpression of CDK5 or p35 mediates cell invasion in pituitary adenomas, which is a common type of neuroendocrine neoplasm [42]. The CDK5–p35 complex plays a regulatory role in the migration of endothelial cells, which might be responsible for angiogenesis [56]. We previously found that CDK5 is responsible for thyroid cancer growth through signal transducer and activator of transcription 3 (STAT3) activation [57]. Although most of the identified CDK5 functions in cancer biology are oncogenic, opposite results were shown in gastric cancer [58,59]. Regarding prostate cancer, our previous findings with in vitro, in vivo, and clinical evidence demonstrated that CDK5 promotes prostate cancer growth through activation of various downstream signaling pathways [8,40,60]. Prostate cancer is one of the most common cancer types in men [61]. Prostate cancer progresses slowly and, in some cases, can be malignant and cause metastasis to other tissues. Age is one of the highest risk factors for prostate cancer, with around 85% of all cases diagnosed in those aged over 65 years and an estimated incidence of only 0.1% in those aged under 50 years [62]. Prostate-specific antigen (PSA), secreted by prostatic epithelial cells, is a serum indicator used to diagnose the incidence of prostate cancer [30]. PSA testing is a sensitive way to detect the tumor growth rate in prostate cancer patients [63]. The levels of androgen and its receptor (androgen receptor, AR) play essential roles in the early development of prostate cancer [40,64,65,66]. One of our previous studies indicates the involvement of CDK5 in prostate cancer growth through negatively regulating p21^CIP1^, a member of the CDK inhibitor family containing conserved N-terminal regions for CDK binding, which suppresses cell cycle progression [67]. Our results suggest that CDK5 decreases p21^CIP1^ protein levels by reducing p21^CIP1^ protein stability via proteasome-dependent degradation in cancer cells [60]. Subsequently, CDK2 involvement was evaluated by CDK2 knocking down in a xenograft model to clarify the CDK5 contribution beyond CDK2 interference since p21^CIP1^ is also a target of CDK2. The results demonstrated that CDK5 still promotes tumor growth under CDK2 inhibition in a prostate cancer cell line in a 22Rv1-derived xenograft model. Interestingly, CDK5-dependent p21^CIP1^ degradation resulted in subsequent CDK2 activation in the nucleus, which promotes prostate cancer growth. Conversely, CDK5 knockdown in a xenograft mouse model increased the p21^CIP1^ protein level and retarded tumor growth. Therefore, CDK5 directly targets nuclear p21^CIP1^ protein and activates CDK2, which represents an important role in the regulation of the cell cycle in the G1-S phase, and therefore, tumor growth is promoted [60]. This is the first piece of evidence indicating that CDK5 is able to manipulate the activity of its sibling, CDK2, which implies tight involvement in cell cycle control. In addition to cell growth, we also claimed that CDK5 is responsible for the apoptosis of prostate cancer cells under the status of drug-triggered “hyperactivation,” which is similar to in AD [53]. These pieces of evidence imply that CDK5 is reasonably sensitive to the control of both prostate cancer survival and growth. In addition to growth regulation, CDK5 has been known as a regulatory protein that controls cytoskeletal remodeling, cell polarity, cell motility, and metastasis of prostate cancer cells [68]. CDK5 participates in actin polymerization, microtubule remodeling, integrin activation, and cellular adhesion, which correlates to its roles in regulating the invasion and motility of cancer cells [68,69,70,71]. The high expression level of p35 has been observed in human metastatic prostate cancer. The blocking of CDK5 activity with its siRNA or Roscovitine resulted in structural changes in the microtubule cytoskeleton, loss of cellular polarity, and loss of motility. These results demonstrated that CDK5 activation is significantly correlated with motility and metastatic activity in prostate cancer cells [68]. The multiple biological processes of CDK5 with neuronal and non-neuronal events are summarized in Figure 1, including gene expression [72], cell migration [73], androgen production [25], apoptosis, and various pathological processes in the different type of cancers [1,74].

## 5. CDK5 and Apoptosis of Prostate Cancer Cells

Apoptosis is known as programmed cell death and may occur in several processes such as cell cycle, immune response against bacterial infections, embryonic development, and chemical-induced cell death [75]. CDK5 regulated by p35 or p25 may trigger many different types of biological responses, including apoptosis of neurodegenerative cells or cancer cells [74,76]. Our previous results demonstrated that hyperactivation of CDK5 would cause apoptosis of prostate and cervical cancer cells [3,53,77,78]. On the other hand, drugs, biological agents, and other components have been commonly used for inducing apoptosis and used in the treatment of cancers. Roscovitine is a common pan-CDK inhibitor and disturbs the cell cycle process through inhibiting DNA synthesis. Therefore, it can be considered as a potential drug for treating cancers [79]. Roscovitine triggers apoptosis of cancer cells by increasing the expression level of p53 while decreasing anti-apoptotic proteins [80,81,82]. We also found a significant decrease in the growth of LNCaP prostate cancer cells as well as in a tumor xenograft animal model after Roscovitine treatment [83]. In addition, under stress conditions such as drug treatment, hypoxia, or immune response, p35- or p25-mediated CDK5 hyperactivation causes cell apoptosis [3,53,78]. Our previous results demonstrated that retinoic acid (RA) plays an essential role in the apoptosis of human hepatoma cells through the direct regulation of p21 expression [84]. We also illustrated that RA induces apoptosis in cervical cancer cells through the hyperactivation of CDK5 [78]. In our previous studies, we showed that CDK5 regulates the apoptosis and proliferation of prostate cancer cells [3,53,77], and that RA treated prostate cancer cells may either induce p35 cleavage into p25 to result in overactive CDK5 activity in a high dose treatment [3] or upregulate CDK5 expression as well as subsequent p27 expression in a low dose treatment [77]. Both the above findings indicate that RA treatment significantly decreases the cell proliferation of human prostate cancer cells. Moreover, Calpeptin, an inhibitor of the p35 protein cleavage enzyme Calpain [53], significantly reverses RA-induced p25 overactivation of CDK5 while p35 protein expression is maintained in human prostate cancer cells and RA-triggered apoptosis is also prevented [3]. Our results also showed that knockdown of CDK5 blocks RA-induced apoptosis in human prostate cancer [3]. Taken together, our data suggest that RA as a trophic factor derived from Vitamin A induces apoptosis of prostate cancer through CDK5 overactivation [3,84,85].

Our previous data also showed that Digoxin, a Digitalis member, reduces the proliferation of prostate cancer cells. Increased intracellular Ca^2+^ was proven to be an important factor in Digoxin-triggered apoptosis of prostate cancer cells [53]. Digoxin induces intracellular Ca^2+^ and prostate cancer cell toxicity [86]. Our previous study demonstrated that Digoxin treatment increases the intracellular Ca^2+^ level and results in decreased proliferation of prostate cancer cells [53]. Besides this, Digoxin treatment causes p25 formation and CDK5 overactivation. The inhibition of p35 cleavage (p25 formation) by Calpeptin, as described previously, prevents Digoxin-triggered apoptosis of prostate cancer cells. These data suggest that p25 formation is essential to Digoxin-triggered apoptosis of prostate cancer cells. Furthermore, treatment with siRNA of CDK5 or inhibitors reduced Digoxin-triggered prostate cancer cell death, suggesting that CDK5 plays an essential role in Digoxin-triggered prostate cancer cell death. We demonstrated the expression and biological functions of CDK5, p35, and p25 in prostate cancer cells for the first time [53].

## 6. CDK5 and AR in Prostate Cancer

**Figure 2:**
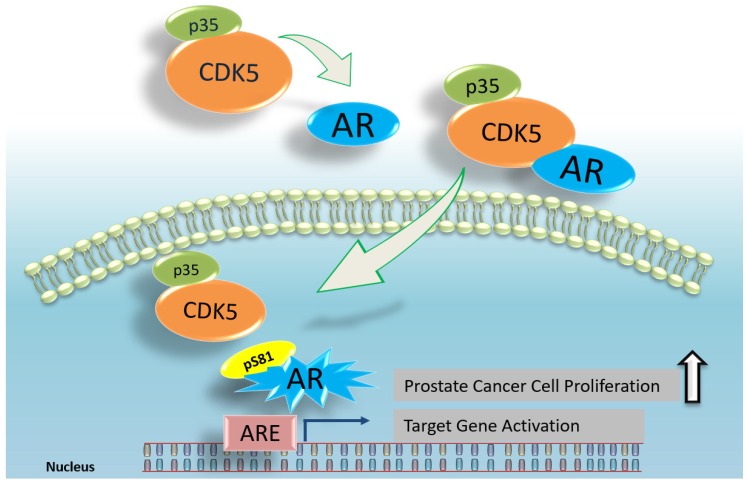
A schematic representation of the regulation of androgen receptor (AR) protein by the interaction of CDK5 and p35. CDK5 and p35 regulate AR by direct biochemical interaction. CDK5 increases the stabilization and transcriptional activation of the AR protein. The consequence of this regulation is the promotion of prostate cancer cell growth.

CDK5 is an essential member of the cyclin-dependent kinase family and was not previously believed to participate in the cell cycle process. However, more and more evidence has recently appeared indicating its correlation with cell-cycle-related proteins. CDK5 and other kinases CDK1 and CDK9 can phosphorylate the AR Ser-81 site [40,50,87]. Our study showed the increased phosphorylation of AR on the Ser-81 site, even in androgen-independent LNCaP prostate cancer cells [88]. This demonstrated that CDK5 may phosphorylate AR via direct biochemical interaction in prostate cancer cells by manipulation of CDK5 with overexpression, knockdown, and drug-targeted inhibition [40]. AR is degraded by the ubiquitin–proteasome pathway [89]. The ubiquitin–proteasome degradation plays a critical role in the stage of transcriptional regulation [90], and the ubiquitin–ligase proteins become important regulators of the steroid receptor family [91]. To identify the effect of the overexpression of CDK5 and p35 on the stabilization of AR protein, we used a protein synthesis inhibitor blocking cellular protein synthesis to observe the degradation of the existing protein. The results indicated that overexpression of CDK5 and p35 decelerates the degradation of AR protein in prostate cancer cells while CDK5 overexpression cannot help the degradation of S81A mutant AR. These results suggest that CDK5 plays an essential role on AR stabilization through Ser-81 phosphorylation. We also found that the knockdown of CDK5 by its siRNA decreased the expression level of nuclear AR localization. Besides, it has been reported that p35 can translocate into the nucleus in prostate cancer cells and possibly regulates nuclear CDK5 and AR activation [92]. These results suggest that CDK5 plays a regulatory role not only in AR stability but also in AR subcellular localization. Interestingly, in the absence of androgen, CDK5 did not affect the transcriptional activity of the AR protein. This result suggests that CDK5 activation plays an important role in AR transactivation in prostate cancer cells in an androgen-dependent manner. In addition, we identified that treatment with Roscovitine, as a pan-CDK inhibitor, decreased the nuclear localization of AR protein in prostate cancer cells, while the cytosolic accumulation of AR protein is increased in prostate cancer cells. In this case, CDK5 activity was significantly affected by Roscovitine treatment. It suggests that Roscovitine prevents AR translocation in prostate cancer cells. Besides, Roscovitine reduces the secreted PSA protein level and ARE activity in LNCaP prostate cancer cells. Our results demonstrated that Roscovitine treatment significantly suppressed AR protein activation, AR subcellular translocation, transcriptional activity, and cell proliferation in prostate cancer cells, which was strongly correlated to CDK5 actions [83]. These results suggest that AR can be a potential target of CDK5 inhibition in regulating the growth of prostate cancer cells, and new small molecular compounds, like Dinaciclib, can be potentially developed as a potential treatment for prostate cancer. Furthermore, our study demonstrated that CDK5 and p35 are positively correlated with the in vitro and in vivo growth of prostate cancer cells through AR regulation. According to these observations, we believe that CDK5 may also play an important role in the clinical events of prostate cancer progression. To support this hypothesis, AR-positive prostate carcinoma specimens were collected, and the results showed that CDK5 and p35 protein levels have a positive correlation with AR protein levels, while the expression levels of AR, p35, and CDK5 were higher in tumor tissue compared with adjacent normal tissue [40]. This clinical finding suggests that CDK5 and p35 certainly play crucial roles in the development of prostate cancer. These data provide the first confirmation of the connection between CDK5 and AR in prostate cancer cells and prostate carcinoma specimens [40], suggesting that phosphorylation of AR by direct interaction with CDK5 protects AR protein against degradation by the ubiquitin–proteasome mechanism. Altogether, it suggests that p35 is also critical to CDK5 and AR regulation and their functions. An illustration of CDK5 and AR regulation in prostate cancer cells is shown in Figure 2.

## 7. CDK5-STAT3-AR in Prostate Cancer

Our previous results indicated that CDK5 increases prostate cancer growth through the activation of AR protein and the CDK5–p35/AR complex translocates into the nucleus and promotes prostate cancer growth (Figure 2) [40]. Several studies have demonstrated that CDK5 activity modulates the signal transducer and activator of transcription 3 (STAT3) protein through Ser-727 phosphorylation in neurons [93,94], head and neck squamous cell carcinoma [95], and liver cancer cells [96]. CDK5 prohibits DNA damage with STAT3 interaction in cancer cells [74,97]. Our previous study also showed that CDK5 regulates thyroid cancer cell proliferation through the activation of STAT3 on Ser-727 phosphorylation [57]. Since CDK5 regulates both STAT3 and AR activation, it is important to further understand the detailed mechanism of this regulation in prostate cancer cells. We found that CDK5 inhibition decreases phosphorylation of STAT3 on Ser-727. Overexpression of p35 increases CDK5–STAT3 interaction as well as Ser-727 phosphorylation. We also analyzed the levels of CDK5, p35, and STAT3 proteins in clinical samples and the results showed that CDK5 expression was positively correlated with STAT3 Ser-727 phosphorylation, which suggested that the phosphorylation of STAT3 plays an essential role in regulating prostate cancer progression and the Gleason score, in addition to similar findings of STAT3 in breast cancer [98]. Interestingly we found that STAT3 acts as a co-activator in the interaction of CDK5 and AR by using the strategy of monitoring mutual interaction among the phospho-site mutants [8]. Our study was the first to demonstrate the interaction between CDK5 and STAT3 Ser-727 phosphorylation in prostate cancer cells and regulates cancer growth [8]. Based on our results, CDK5 activation promotes the growth of prostate cancer cells by direct interaction with STAT3 through Ser-727 phosphorylation, while CDK5-mediated STAT3 activation contributes to full AR transactivation in addition to CDK5-dependent Ser81-AR regulation, as described previously. Taken together, these findings portray the essential role of CDK5–STAT3–AR regulation in the progression of prostate cancer led by CDK5 activation [8].

## 8. The Relationship between CDK5 and Castration-Resistant Prostate Cancer

Androgen deprivation treatment (ADT) is an essential therapeutic option for prostate cancer. Many prostate cancer patients are receiving standard ADT, and this treatment shows a better outcome for patient recovery within three years [99,100]. However, after ADT, there is a high possibility that many patients will develop castration-resistant prostate cancer (CRPC) [101]. Tumor cells transform themselves from the androgen-dependent phase to the androgen-independent phase, and AR is a critical element in this transition. Recent studies have reported that CRPC is strongly related to AR regulation [102,103]. Once prostate cancer progresses to the androgen-independent phase, the hormone-refractory treatment fails, although the molecular mechanisms of this transition remain unclear. To address this prostate cancer mystery, we established an androgen-insensitive LNCaP cell subline, LNCaPdcc, to understand how the AR protein level is regulated in prostate cancer cells in an androgen-free environment [88]. Our data showed that the S phase distribution of LNCaPdcc cells was markedly higher than that of parental LNCaP cells. Cell cycle distribution is important to cancer cell growth, and the levels of cell-cycle-related proteins (CDK1, cyclin A, cyclin B1, and cyclin D1) in LNCaPdcc cells were lower than those in parental LNCaP cells, suggesting that LNCaPdcc cells have a slower growth pace (doubling time) than parental cells. However, higher protein levels of AR (even nuclear AR) and PSA were observed in LNCaPdcc cells, indicating that some mechanisms were in place to support AR activation in LNCaPdcc cells without the presence of androgen [88]. Previous findings have shown a correlation between AR and human epidermal growth factor receptor 2 (Her2) in androgen-dependent prostate cancer cells, and Her2/ErbB3 activation stabilizes AR protein [104]. Our data also showed that the AR protein level was associated with Her2 activation, and Her2 and its partners ErbB3 and ErbB4 showed higher expression levels in LNCaPdcc cells. Inhibition of Her2 induced AR protein degradation and significantly reduced phosphorylation of AR Ser-81. These data indicate that Her2 activation is important to AR Ser-81 phosphorylation and plays an essential role in AR stabilization in the circumstance of androgen deprivation [88]. Our results demonstrate that LNCaPdcc cells showed much slower growth, lower expression of cell-cycle-related proteins, and neuroendocrine morphology. Importantly, our data showed that Her2 inhibition increases AR degradation in LNCaPdcc cells compared to parental LNCaP cells. These findings propose that Her2 regulation has a crucial role in prostate cancer cell growth by affecting AR protein stability in either AR-positive prostate cancer cells or androgen-insensitive prostate cancer cells [88,105]. Herceptin is a humanized anti-Her2 monoclonal antibody that is widely used for the treatment of Her2-positive breast cancer patients [106,107]. In our previous study, we investigated the effects of radioimmunotherapy against Her2 in DU145, a common CRPC cell line. Treatment with isotope Re-188 labeled Herceptin (Re-H) significantly reduced the proliferation of DU145 cells in dose- and time-dependent manners compared to the Herceptin-treated group [108]. Re-H treatment significantly retarded tumor growth with significant apoptosis. Importantly, the levels of p35 and CDK5 protein were dramatically decreased after Re-H treatment in both in vitro and in vivo models [108]. On the other hand, our previous study demonstrated that CDK5 inhibition reduced tumor formation with phosphorylation of STAT3 on Ser-727, and it has been reported that Her2 is an upstream protein of CDK5 in thyroid carcinoma cells [57]. Taken together, these observations indicate that Her2 activation plays an important role in AR Ser-81 phosphorylation and AR stabilization while treatment with Re-188 labeled Herceptin (Re-H) effectively decreases the p35 protein expression level. These findings elucidate the potential regulation between CDK5 and Her2. According to this evidence, we propose that Her2 and CDK5–p35 interaction with AR regulation might be relevant to future CRPC diagnosis and treatment.

## 9. Conclusion and Future Perspectives

**Figure 3:**
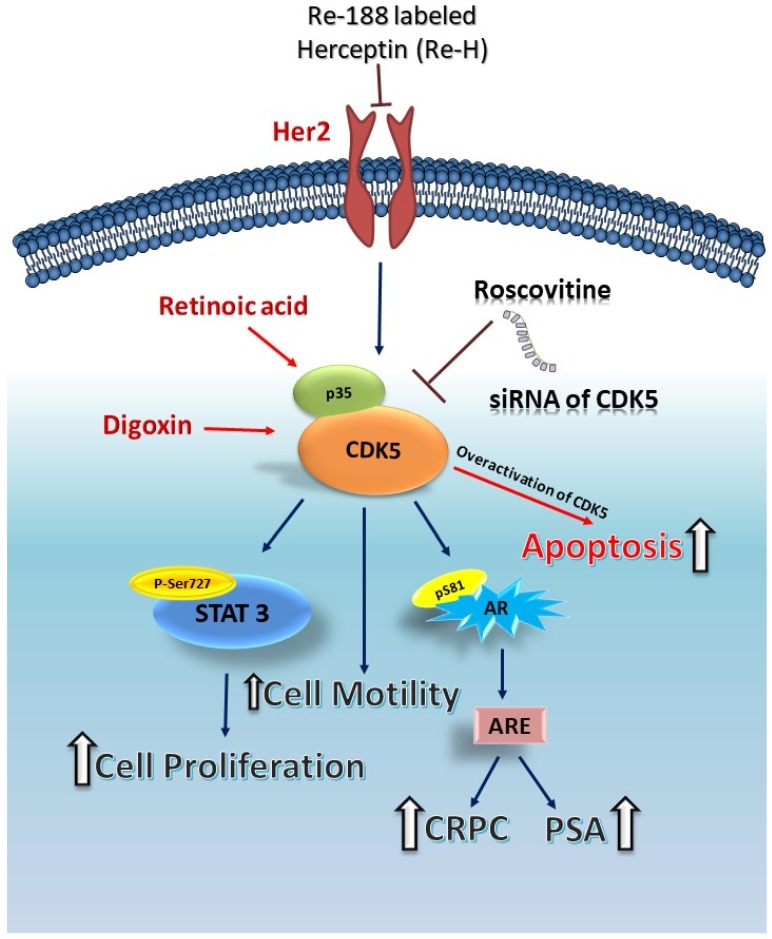
A schematic summary of the regulatory role of CDK5 in prostate cancer. Re-188 labeled Herceptin (Re-H) targets the Her2 receptor and reduces the expression level of p35. CDK5 activates signal transducer and activator of transcription 3 (STAT3) and AR proteins at the specific phosphorylation site. Activation of STAT3 or AR causes prostate cancer proliferation and castration resistance of prostate cancer. Blocking of CDK5 by Roscovitine or small interfering RNA (siRNA) decreases the cell proliferation ability and prostate-specific antigen (PSA) level. CDK5 also plays a regulatory role in apoptosis of prostate cancer cells. Digoxin and Retinoic acid trigger p35 or p25 activation and cause overactivation of CDK5, resulting in apoptosis.

CDK5 is an essential kinase in postmitotic neurons and participates in the development of the central nervous system. CDK5 hyperactivation is important to the development of neurodegenerative diseases. CDK5 is involved not only in the central nervous system, but also in various biological and pathological processes such as gene expression, migration, apoptosis, and proliferation of prostate cancer, which is the most common type of cancer in men. The activator protein of CDK5, p35, is necessary to the activation of CDK5 protein in both the central nervous system and cancer tissues. The latest findings show that CDK5 is related to the pathophysiology of cancer-related pain [109] and type II diabetes [110]. Besides, CDK5 plays a vital role in DNA damage repair (DDR) [41,97,111,112], while it also plays a crucial role in regulating the activation of different tumor suppressor genes [20,111,113]. AR is a regulatory protein essential to prostate cancer growth. Our findings suggest that CDK5 signaling plays a vital role in AR activation and controls the proliferation of prostate cancer cells [40]. Knockdown of CDK5 affects AR nuclear localization and stability, suggesting that CDK5 contributes to prostate cancer growth through regulating AR subcellular localization. On the other hand, we reported that CDK5 promotes the proliferation of prostate cancer cells through a direct protein–protein interaction with STAT3 protein. CDK5–STAT3 interaction results in AR activation with a triangle connection. This innovative finding demonstrates that CDK5–STAT3–AR regulation is essential for the regulation of prostate cancer progression. Besides, CDK5 and p35 were found to be associated with metastasis [68] in our study of p35 overexpression in human metastatic prostate cancer [8]. Concerning cell fate, our studies demonstrated that CDK5 plays a vital role in the regulation of apoptosis in prostate cancer [3,53]. Blocking of CDK5 by siRNA or inhibitor resulted in decreased PSA levels, AR protein activation, AR subcellular translocation, and cell proliferation in prostate cancer cells. On the other hand, drug-triggered CDK5 hyperactivation resulted in the apoptosis of prostate cancer cells. Castration-resistant prostate cancer commonly occurs after androgen deprivation treatment when prostate cancer cells transform themselves from the androgen-dependent phase to the androgen-independent phase. In this stage, AR is a crucial element for this transition and can be regulated by CDK5. We identified that CDK5 plays an essential role in the regulation of CRPC, as shown in our androgen deprived LNCaP subline, LNCaPdcc. Taken together, these results indicate that CDK5 is a vital kinase in prostate cancer and could be an important drug target. Therefore, the potential of small molecular inhibitors or peptides to block CDK5 kinase activity have attracted scientists’ attention in the past decade. In this paper, we provided a relevant literature review of the role of CDK5 in the development of prostate cancer, and this is summarized in Figure 3. Since CDK5 has recently become a potential target for anti-cancer drug discovery [1,111], CDK5 and p35 can be considered as potential diagnostic markers as well as therapeutic targets for multiple diseases and different types of cancers, especially prostate cancer, in the near future.

## Abbreviations

CDK5	Cyclin-dependent kinase 5
CNS	Central nervous system
AR	Androgen receptor
siRNA	Small interfering RNAs
AD	Alzheimer’s disease
StAR	Steroidogenic acute regulatory
STAT3	Signal transducer and activator of transcription 3
PKC	Protein kinase C
DBD	DNA binding domain
AREs	Androgen responsive elements
IGF	Insulin-like growth factor
RA	Retinoic Acid
ADT	Androgen deprivation treatment
CRPC	Castration-resistant prostate cancer
